# Suppression of pyruvate dehydrogenase kinase-2 re-sensitizes paclitaxel-resistant human lung cancer cells to paclitaxel

**DOI:** 10.18632/oncotarget.16991

**Published:** 2017-04-10

**Authors:** Hong Sun, Anyou Zhu, Xiang Zhou, Fengchao Wang

**Affiliations:** ^1^ Department of Clinical Laboratory Science, The First Affiliated Hospital of Bengbu Medical College, Bengbu, China; ^2^ Department of Nuclear Medicine, Ren Ji Hospital, School of Medicine, Shanghai Jiao Tong University, Shanghai, China

**Keywords:** glycolysis, pyruvate dehydrogenase kinase-2, drug resistance, NSCLC

## Abstract

Despite impressive initial clinical responses, the majority of lung cancer patients treated with paclitaxel eventually develop resistance to the drug. Pyruvate dehydrogenase kinase-2 (PDK2) is a key regulator of glycolysis and oxidative phosphorylation, and its expression is increased in a variety of tumors. In this study, the role of PDK2 in mediating paclitaxel resistance in lung cancer cells was investigated using biochemical and isotopic tracing methods. Increased expression of PDK2 was observed in paclitaxel-resistant cells ascompared totheir parental cells. Down-regulation of PDK2 usingsiRNA increased the sensitivity to paclitaxel of resistant lung cancer cells. Targeting paclitaxel-resistant cells throughPDK2 knockdown was associated with reduced glycolysis rather than increased oxidative phosphorylation (OXPHOS). Moreover, combining paclitaxel withthe specific PDK2 inhibitor dichloroacetate had a synergistic inhibitory effect on the viability of paclitaxel-resistant lung cancer cells. These results indicate that paclitaxel-induced expression of PDK2 serves as an important mechanism for acquired paclitaxel resistance of lung cancer cells. They also highlight the importance of PDK2 for potential therapeutic interventions in patients who have developed a resistance to paclitaxel.

## INTRODUCTION

Non-smallcell lung cancer (NSCLC) is one of the most common malignant tumors and a leading cause of mortality worldwide. Chemotherapy is a crucial strategy for advancedstage NSCLC. Paclitaxel (Taxol), which targets microtubules of cancer cells, has been widely used in cancertreatment [1, 2]. Paclitaxel disrupts the dynamic equilibrium between soluble tubulin dimers and their polymerized form to stabilize the microtubule structure. In addition, paclitaxel is an effective inhibitor of chromosomal replication by obstructing cancer cells in the late G2 or mitotic phases [3]. However, the efficiency of paclitaxel-based chemotherapy is limited by the development of acquired resistance. Increasedexpression of multidrug resistant proteins and anti-apoptotic proteins isthe main cause of paclitaxel resistance [4]. However, the specific molecular mechanisms involved in paclitaxel resistance are complex and not completely understood.

In 1920s, Otto Warburg demonstrated that cancer cells exhibit increased glycolysis, even when oxygen is abundant. This phenomenon of enhanced aerobic glycolysis is known as the Warburg effect [5, 6]. Cancer cells, unlike normal cells, often use aerobic glycolysis instead of mitochondrial oxidative phosphorylation (OXPHOS). The Warburg effect is closely associated with drug resistance in cancer cells. Agents that target glycolysis or OXPHOS have shown promising efficacy in overcoming dug resistance [7–9].

Pyruvate dehydrogenase kinase (PDK) is one of the key regulators of glycolysis and oxidative phosphorylation. PDK phosphorylates pyruvate dehydrogenase (PDH) to inhibit the conversion of pyruvate to acetyl-CoA, and plays a key role in oxidative phosphorylation, proliferation, and tumor maintenance of cancer cells [10–18]. However, the correlation between PDK expression and paclitaxel resistance of cancer cells is still unclear. In this study, we investigated the molecular mechanisms involved in the paclitaxel-resistance of NSCLC cells, and the relationship betweenPDK2 and paclitaxel resistance. Our data show that paclitaxel-resistant NSCLC cellsexhibit increased expression of the PDK isoform 2 (PDK2) compared with their parental cells. Suppression of PDK2 or use of the PDK inhibitor dichloroacetate (DCA) increase sensitivity of NSCLC cells to paclitaxel. Importantly, combination of paclitaxel and DCA hasa synergistic inhibitory effect on theviabilityof NSCLC cells. Together, our results indicate that PDK2 plays an important role in paclitaxel resistance of NSCLC cells, and therefore serves as a promising therapeutic target for overcoming paclitaxel resistance in NSCLC.

## RESULTS

### Selection and characterization of paclitaxel-resistant A549-R cells

A549 cells were treated with increasing concentrations of paclitaxel in culture medium for selection of paclitaxel-resistant cells. After 6 months, one resistant cell clone (A549-R) was obtained from the A549 parental cell line and was used for subsequent experiments.

**Figure 1:**
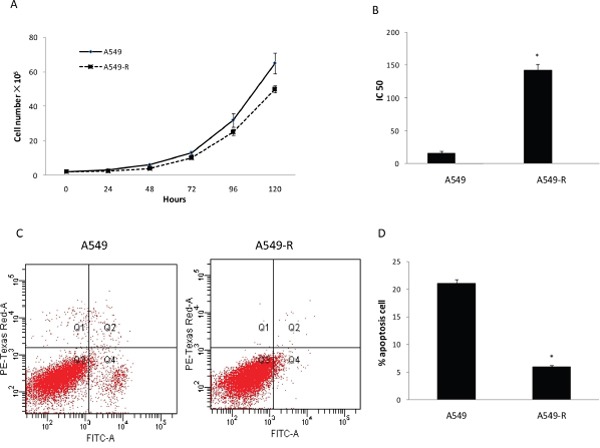
Characterization of paclitaxel-resistant A549-R cells **(A)** Viability of A549 and A549-R cells determined by cell counting. **(B)** IC_50_ for paclitaxel in A549 and A549-R cells after paclitaxel treatment for 48 h. (**C** and **D**) Apoptosis in A549 and A549-R cells treated with 10 nM paclitaxel for 48 h by flow cytometry using Annexin V/PI staining and its representation in bar format. (*, P<0.05).

As shown in Figure 1A, A549-R cells displayed a decreased growth rate compared with A549 cells. The CCK8 assay was performed to confirm the resistance to paclitaxel. The IC_50_ of paclitaxel was 142 ±9.5 nM in A549-R cells and 16 ±2.8 nM in A549 cells (Figure 1B). To compare the survival capacity of A549-R and A549 cells, the number of apoptotic cells was measured by flow cytometry. A549-R cells displayed a decreased apoptosis compared with A549 cellsincubated 24 h with 15 nM paclitaxel (Figure 1C, 1D).

**Figure 2:**
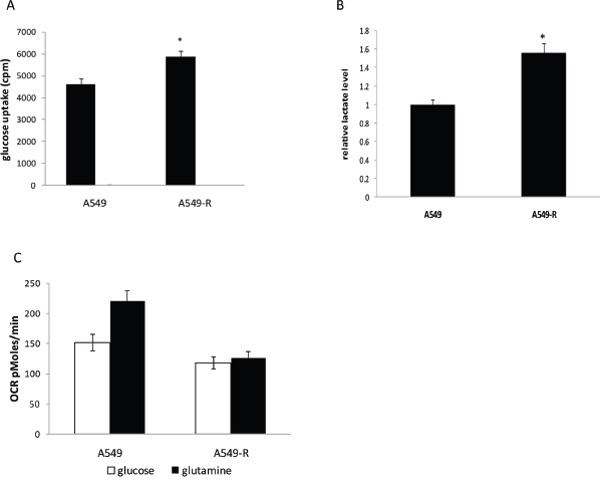
Changes in glycolysis in paclitaxel-resistant cells **(A)** Comparison of glucose uptake in A549 and A549-R cells. **(B)** Lactate production rate detected in A549 and A549-R cells. **(C)** OCR in A549 and A549-R cells using glucose or glutamine as the carbon source. (*, P<0.05).

Glucose glycolysis and mitochondrial function in A549 and A549-R cells were assessed. A549-R cells showed a significant increase in glucose uptake (Figure 2A) and lactate production (Figure 2B) compared with A549 cells. Oxygen consumption rate (OCR) is linked to respiration and can be used as a surrogate marker for mitochondrial function. To estimate the mitochondrial function, the OCR was measured using glucose or glutamine as the carbon source. As shown in Figure 2C, A549-R cells displayed significantly reduced oxidative ability in the presence of glucose or glutamine as a carbon source.

### Inhibition of PDK2 restores A549-R cell sensitivity to paclitaxel

**Figure 3:**
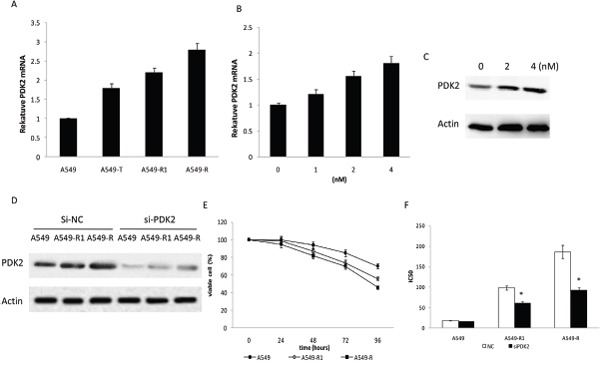
Increased PDK2 expression in paclitaxel-resistant cells **(A)** Comparative analysis of mRNA levels of PDK2 in A549, A549-T, A549-R1 and A549-R cells. **(B)** PDK2 mRNA levels detected by qRT-PCR under increasing concentrations of paclitaxel in A549 cells. **(C)** Western blot performed with antibodies against PDK2 and actin on cell lysates from A549 cells treated with increasing concentrations of paclitaxel for 48 h. **(D)** Western blot performed with antibodies against PDK2 on cell lysates from A549, A549-R1 and A549-R cells transfected with scrambled siRNA (NC) or PDK2 siRNA. **(E)** Cell survival rate determined by cell counting after treatment of A549, A549-R1 and A549-R cells with PDK2 siRNA. **(F)** IC_50_ for paclitaxel in A549,, A549-R1 and A549-R cells after treatment for 48 h with paclitaxel. Inhibition of PDK2 restored A549-R cell sensitivity to paclitaxel. (*, P<0.05).

The above results suggest that the paclitaxel resistance in A549 cells is associated with increased glycolysis and suppressed oxidative phosphorylation. As PDK2 is one of the key regulators of glycolysis and oxidative phosphorylation, its expression was examined in A549-T cells (A549 cells treated with 4 nM paclitaxel for 48 h), A549-R and A549-R1 cells (A549-R and A549-R1 cells have different paclitaxel resistance), and A549 cells(treated with 0 nM paclitaxel) to evaluate the role of PDK2 in mediating paclitaxel resistance. PDK2 mRNA and protein levelswere markedly increased in A549-R cells compared to A549 cells (Figure 3A-3D). In addition, paclitaxel increased PDK2 expression in a dose-dependent manner in A549 cells (Figure 3B, 3C). These results indicate that PDK2 plays animportant role in paclitaxel resistance. Therefore, we investigated the effect of PDK2 suppression on paclitaxel sensitivity. As the expression of PDK2 was increased in A549-R and A549-R1 cells, we hypothesized that the down-regulation of PDK2 by siRNA might re-sensitize A549-R cells to paclitaxel. To this end, PDK2 was suppressed with siRNA in A549-R and A549-R1cells (Figure 3D), and the cells were then treated with different concentrations of paclitaxel. As shown in Figure 3E, cell survival ratewasdecreasedin A549, A549-R, and A549-R1 cells with suppressed PDK2. Down-regulation of PDK2 inhibited survival rate of A549-R cellsmore than the less resistantA549-R1 cells and their parental A549 cells. A549-R cells showed increased sensitivity to paclitaxel compared to A549-R1 and their parental A549 cells(Figure 3F). These results indicate that paclitaxel resistance is associated with increased PDK2 expression and that knockdown of PDK2 may re-sensitize A549-R andA549-R1cells to paclitaxel.

### Targeting A549-R cells by PDK2 suppression is attributed to inhibited glycolysis rather than increased OXPHOS

**Figure 4:**
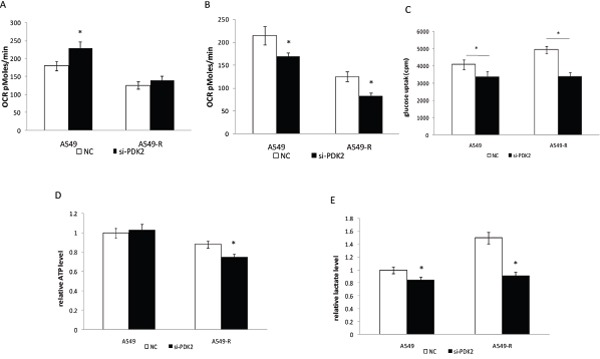
Inhibition of glycolysis in A549 and A549-R cells by PDK2 RNAi **(A)** OCR in A549 and A549-R cells treated by siRNA-PDK2 for 48 h using glucose as the carbon source. **(B)** OCR in A549 and A549-R cells treated by siRNA-PDK2 for 48 h using glutamine as the carbon source. **(C)** Glucose uptake in A549 and A549-R cells transfected with PDK2 siRNA. Cells were incubated in DMEM with 18F-FDG for 1 h and radioactivity was measured using the well γ-counter. **(D)** Quantification of ATP levels in cells 48 h post-transfection with PDK2-siRNA. **(E)** Lactate production rate determined in A549 andA549-R cells 48 h post-transfection with PDK-siRNA. (*, P<0.05).

We further assessed whether suppression of PDK2 re-sensitizes A549-R cells to paclitaxel by suppression of glycolysis or activation of OXPHOS. As shown in Figure 4A, knockdown of PDK2 increased OCR by 10 ± 3.8% in A549-R cells, and 28.5 ± 5.5% in A549 cells (*P*<0.01, compared with untreated control). When glutamine was used as a carbon source, PDK2 siRNA inhibited glutamine oxidation through an unclear mechanism (Figure 4B). Furthermore, knockdown of PDK2 inhibited glutamine oxidation more effectively in drug resistant cells.

**Figure 5:**
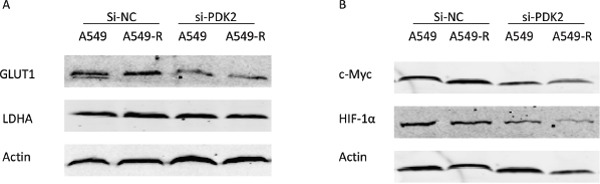
Protein expression changes post-transfection with PDK2-siRNA **(A)** Effects of PDK2-siRNA on the expression of GLUT1 and LDHA 48 h post-transfection. **(B)** Effects of PDK2-siRNA on the expression of c-Myc and HIF-1α 48 h post-transfection. (*, P<0.05).

The Warburg effect is closely associated with drug resistance in cancer cells [17, 18]. Glucose uptake and lactate generation ability areimportant indicatorsof cell glycolytic activity. In our study, we found that knockdown of PDK2 reduced glucose uptake and lactate production inA549-R cells (Figure 4C, 4D). Next, we analyzed the effect of PDK2 suppressionon ATP production and glycolysis. PDK2 suppression decreased ATP generation in A549-R cells, but failed to reduce ATP production in A549 cells (Figure 4E). These results indicate that targeting A549-R cells by PDK2 knockdown is attributed to inhibited glycolysis rather than increased OXPHOS in paclitaxel-resistant cells. To elucidate the mechanism of reduced glucose uptake and lactate production, the effect of PDK2 suppressionon the expression of glucose transporter1 (GLUT1) and the glycolytic enzyme lactate dehydrogenase A (LDHA) was investigated (Figure 5A). A significant decrease in GLUT1 expression was observed following PDK2 knockdown in A549 and A549-R cells; however, expression of LDHA was not changed after PDK2 knockdown in A549 cells or A549R cells.

Next, we examined the effect of PDK2 knockdown on the expression of c-Myc and HIF-1α, two major transcription factors that regulate the expression of GLUT1. Interestingly, c-Myc protein levels were not changed after PDK2knockdown in A549 cells, but PDK2 suppression reduced the c-Myc levels compared to untreated control in A549-R. HIF-1α levels were decreased after treatment in A549 and A549-R cells. In addition, PDK2 suppression decreased HIF-1α in A549-R cells compared to A549 cells. These results indicate that PDK2suppression inhibits glycolysis more significantly in paclitaxel resistant cells.

### Combination of paclitaxel with dichloroacetate(DCA) shows a synergistic inhibitory effect on A549 cells

**Figure 6:**
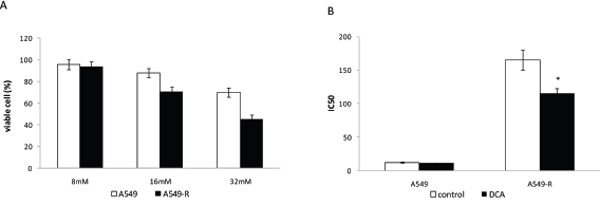
DCA resensitizes paclitaxel-resistant cells to paclitaxel **(A)** Viability of A549 and A549-R cells treated with various concentrations of DCA for 48 h. **(B)** IC_50_ for paclitaxel in A549 and A549-R cells after treatment for 48 h with 0 or 10 mM DCA. (*, P<0.05).

DCA, a small chemical compound, inhibits PDK-mediated inactivation of PDH (pyruvate dehydrogenase) and diverts glucose metabolism from glycolysis towards OXPHOS [13]. As shown in Figure 6A, the effect of DCA on A549 cell viability was assessed. DCA treatment decreased cell viability in a dose-dependent manner in both A549 and A549-R cells. A549-R cells displayed increased sensitivity to DCA compared to A549 cells, consistent with the results of PDK2 knockdown by siRNA.

As down-regulation of PDK2 by siRNA or DCA inhibited the viability of A549-R cells, the effect of combining paclitaxel with DCA on paclitaxel-resistant cells was investigated. In A549-R cells, paclitaxel combined with DCA was markedly more effective in inhibiting cell viability compared with either agent used alone (Figure 6B). These results demonstrate that the combination of paclitaxel with DCA has a greater capacity to decrease the viability of paclitaxel-resistant cells compared to either agent used alone.

## DISCUSSION

In this study, the role of PDK2 in acquired paclitaxel resistance in A549 cells was investigated. In comparison to paclitaxel-sensitive cells, PDK2 expression was increased in paclitaxel-resistant cells. Knockdown of PDK2 increased sensitivity of paclitaxel-resistant cells to paclitaxel. Compared to paclitaxel-sensitive cells, A549-R cells displayed higher sensitivity to the PDK inhibitor DCA. In addition, when compared to single agent therapy, treating cells with the combination of paclitaxel and DCA exhibited an increased inhibitory effect on A549-R cells.

Paclitaxel is a widely used chemotherapeutic agent for the treatment of malignant tumors, including lung cancer. However, the resistance of cancer cells to paclitaxel is an important issue and can lead to subsequent recurrence and metastasis of malignant tumors [19–21]. To date, the specific molecular mechanisms involved in paclitaxel resistance are still poorly understood. Although the Warburg effect is closely associated with drug resistance in cancer cells, the relationship between the Warburg effect and paclitaxel resistance is unclear.

Previous studies have demonstrated that PDK2 plays a critical role in OXPHOS, glycolysis, and tumor maintenance of cancer cells [22, 23]. Suppression of PDK2 increases apoptosis of cancer cells via activated OXPHOS, suggesting that it might be used as a therapeutic strategy for treatment of malignant tumors. However, the role of PDK2 in paclitaxel resistance of cancer cells has not been studied. Our study indicates that paclitaxel increases PDK2 expression in A549 cells by increasing PDK2 transcription. Suppression of PDK2 by PDK2 siRNA or the inhibitor DCA increases sensitivity of A549 and A549-R cellsto paclitaxel. This indicates that the paclitaxel-induced PDK2 expression in paclitaxel-resistant cells may be an adaptive mechanism in these cells to modulate glycolysis and OXPHOS to avoid the paclitaxel-inducedapoptosis. In addition, since PDK inhibition was more effective in suppressing cell growth of the resistant A549-R cells than the parental A549 cells, these results suggest that PDK2 may serve as an effective target for overcoming paclitaxel resistance in patients with lung cancer. To our knowledge, this is the first report to demonstrate the role ofPDK2 in acquired paclitaxel resistance in human lung cancer cells.

Previous studies have indicatedthat the Warburg effect contributes to drug resistance of cancer cells [24]. However, the underlying molecular mechanisms remain largely unclear. The current study demonstrates that the paclitaxelresistance of A549 cells is associatedwith increased glycolysis and suppressed oxidative phosphorylation. Furthermore, our study indicates that PDK2 suppression reversesthe paclitaxel resistance through suppression of glucose glycolysis rather than activation of OXPHOS in paclitaxel resistant cells.

Since inhibition of PDK2 decreased glucose uptake and lactate production, we investigated the effect ofPDK2 knockdown on the glycolytic enzyme LDHA and the glycolytic transporter GLUT1. A significant decrease in GLUT1 expression was observed after PDK2 knockdown in A549 cells. PDK2 knockdown therefore increased glucose uptake and lactate generation, possibly by up-regulation of GLUT1 expression.

HIF-1α and c-Myc are two major oncogenic transcription factors known to regulate metabolism in cancer cells [25–27]. HIF-1α and c-Myc can up-regulate transcription of GLUT1, sustaining the Warburg effect in cancer cells [28, 29]. PDK2 knockdown had no significant effect on c-Myc levels in A549 cells, but strongly inhibited c-Myc expression in paclitaxel resistant 549-Rcells. In addition, in both A549 cells and A549-R cells, PDK2-siRNA decreased HIF-1α levels. Since the effect of PDK2 knockdown was more pronounced in drug resistant cells, these results suggest that PDK2 can target paclitaxel resistant cells to inhibit glycolysis. The combination of paclitaxel with DCA was more effective in killing paclitaxel-resistant cells, compared to either paclitaxel or DCA treatment alone. The synergistic inhibitory effect of the combination therapy indicates that it might represent an effective strategy to overcome the paclitaxel resistance of NSCLC cells.

In summary, the present study shows that PDK2 plays an important role in paclitaxel resistance, with paclitaxel-induced expression of PDK2 serving as an important mechanism for the acquired resistance of human lung cancer cells to paclitaxel. The results of this study provide valuable information for the development of targeted therapies for paclitaxel-resistanceby inhibiting PDK2, and highlight the importance of PDK2 in paclitaxel resistance.

## MATERIALS AND METHODS

### Cell culture and transient transfection

Human lung adenocarcinoma cell line A549 was obtained from the Chinese Academy of Sciences. A549R and A549R1 cells are paclitaxel-resistant clones developed as previously described [19]. Briefly, the parental A549 cellswere exposed to increasing paclitaxel concentrations, and the surviving resistant A549-R cells were cultured in DMEM containing 5 mM glucose supplemented with 10% fetal bovine serum (FBS), sodium pyruvate and L-glutamine. A549 cells treated with 4 nM paclitaxel for 48 h were designated as A549-T cells. Lipo2000 (Invitrogen) was used for transient transfection according to the manufacturer's protocol. Control siRNAand siRNA-PDK2 were purchased from Pharma (China).

### Uptake of 18F-flurodeoxyglucose

Cells were cultured in 12-well culture plates, then detached, washed twice, and subsequently incubated in 500 μl of DMEM containing 4 μCi/mL of ^18^F-flurodeoxyglucose (18F-FDG) for 1 h at 37°C. Pellets were washed twice with ice-cold phosphate buffered saline (PBS). Cell lysates were obtained using 500 μl of 0.1 M NaOH, and then the radioactivity of the whole-cell lysates was assayed using a well γ-counter. The readouts were normalized to corresponding protein amounts (Beyotime).

### Lactate production assay

Cells were seeded onto 6-well plates and transfected with PDK2 siRNA, or treated with DCA. Approximately 48 h after transfection, cells were washed and cultured in serum-free DMEM for 16 h. Lactate levels in the medium were measured using a lactate assay kit (CMA Microdialysis). The readouts were normalized to corresponding protein amounts (Beyotime).

### Western blot analysis

Cells were harvested and lysed in buffer containing 50 mM Tris-HCl, 150 mM NaCl, 2 mM ethylenediaminetetraacetic acid, 1% Triton and 1 mM phenylmethylsulfonyl-fluoride (PMSF; Sigma) for 10 min on ice. Protein concentration was determined by the bicinchoninic acid protein assay. Proteins were separated using sodium dodecylsulfate polyacrylamide gel electrophoresis, and transferred onto nitrocellulose membranes. The membranes were incubated 3 h at 37°C with primary antibodies in PBS with 5% nonfat milk. The following primary antibodies were used: anti-PDK-2 rabbit antibody (1:1000; Abcam) and anti-β-actin antibody (1:2000, Sigma). Membranes were extensively washed with PBS and incubated with secondary anti-rabbit antibody (1:10000; LI-COR Biosciences). After washing with PBS, protein bands were visualizedand analyzed using a Gel DocXR system (Bio-Rad) and IMAGE LABTM software (version 2.0; Bio–Rad).

### Oxygen consumption rate analysis

Oxygen consumption rate (OCR) was measured using the Seahorse XF24 analyzer (Seahorse Bioscience). Cells were seeded in a 24-well culture plate and allowed to attach overnight. Approximately 30 min prior to the assay, culture medium was changed to the Seahorse assay medium (DMEM containing 5 mM glucose or 2 mM L-glutamine) and OCR was measured according to the manufacturer's instructions.

### Cell survival rate CCK-8 assay

Cell survival rate was examined using the Cell Counting Kit-8 (CCK-8; Dojindo Molecular Technologies), which is based on dehydrogenase activity detection in viable cells. Cells were plated in 96-well culture plates at a density of 6000 cells/well. After 24 h, the medium was replaced with DMEM containing 10% FBS and drugs at the indicated concentrations. Following 24 h incubation, 10 μL of CCK-8 solution was added to each well, and cells were further incubated for 2 h. Absorbance was read at 450 nm using a micro-plate reader. Tocalculate IC50, A549 and A549-Rcells werecultured with increasingpaclitaxelconcentrations, and the IC_50_ value was calculated using the GraphPad Prism version 5.0 software.

### Cellular ATP level assay

Relative cellular ATP content was measured using the ATP bioluminescent somatic cell assay kit (Sigma, St. Louis, MO, USA) according to the manufacturer's instructions. Briefly, 2×10^5^ cells were seeded in a 24-well plate. After 24h, cells were washed, centrifuged, and lysed. Lysates were collected, and luminescence was measured using a luminescence reader and normalized to the protein concentration. Measurements were performed in triplicates [9].

### Statistical analysis

Statistical differences between the groups were assessed using two-tailed ANOVA and t-tests. P values <0.05 were considered significant.

## Abbreviations

DCA: dichloroacetate
GLUT1: glucose transporter 1
LDHA: lactate dehydrogenase A
NSCLC: Non-small-cell lung cancer
OCR: Oxygen consumption rate
OXPHOS: oxidative phosphorylation
PDH: pyruvate dehydrogenase
PDK2: Pyruvate dehydrogenase kinase-2
